# Stably Expressed Genes Involved in Basic Cellular Functions

**DOI:** 10.1371/journal.pone.0170813

**Published:** 2017-01-26

**Authors:** Kejian Wang, Vikrant Vijay, James C. Fuscoe

**Affiliations:** Division of Systems Biology, National Center for Toxicological Research, Food and Drug Administration, Jefferson, Arkansas, United States of America; Harvard Medical School, UNITED STATES

## Abstract

Stably Expressed Genes (SEGs) whose expression varies within a narrow range may be involved in core cellular processes necessary for basic functions. To identify such genes, we re-analyzed existing RNA-Seq gene expression profiles across 11 organs at 4 developmental stages (from immature to old age) in both sexes of F344 rats (n = 4/group; 320 samples). Expression changes (calculated as the maximum expression / minimum expression for each gene) of >19000 genes across organs, ages, and sexes ranged from 2.35 to >10^9^-fold, with a median of 165-fold. The expression of 278 SEGs was found to vary ≤4-fold and these genes were significantly involved in protein catabolism (proteasome and ubiquitination), RNA transport, protein processing, and the spliceosome. Such stability of expression was further validated in human samples where the expression variability of the homologous human SEGs was significantly lower than that of other genes in the human genome. It was also found that the homologous human SEGs were generally less subject to non-synonymous mutation than other genes, as would be expected of stably expressed genes. We also found that knockout of SEG homologs in mouse models was more likely to cause complete preweaning lethality than non-SEG homologs, corroborating the fundamental roles played by SEGs in biological development. Such stably expressed genes and pathways across life-stages suggest that tight control of these processes is important in basic cellular functions and that perturbation by endogenous (e.g., genetics) or exogenous agents (e.g., drugs, environmental factors) may cause serious adverse effects.

## Introduction

The development of genome-wide transcriptional profiling techniques has enabled measurement of the expression of tens of thousands of genes in parallel [1]. A consensus has formed that the status of a cell or an organ is, at least partially, a function of gene expression levels [2]. Frequently, investigations using transcriptomics data identify the genes whose expression levels differ between distinct biological conditions; for example, normal verses diseased tissues [3], or perturbed verses unperturbed samples [4]. Genes that are mostly responsible for the difference between the biological states are defined as differentially expressed genes (DEGs), which aid in the development of biomarkers for clinical diagnosis [5] and the understanding of diverse biological mechanisms of diseases [6, 7].

Genes whose expression remains relatively constant across multiple conditions are important in research methodologies and in understanding gene regulation. For example, quantification of often subtle changes in gene expression by PCR is performed through comparison with the quantity of endogenous controls, such as housekeeping genes [8], whose expression does not change under the experimental conditions. Housekeeping genes are involved in basic cell maintenance and, therefore, are expected to be present in all cells and maintain relatively constant expression levels under different experimental conditions. Identification of these genes increases understanding of various structural genomic features and facilitates the accurate measurement of expression of target genes [9]. Recently, a series of studies demonstrated that variability in gene expression can provide important insights into gene regulatory control related to development [10] and pathogenesis [11]. In an analysis of single cell RNA-seq data derived from embryos, those genes with low expression variability across developmental stages were found to have a functional impact on the regulation of basic cellular processes, and less likely to be associated with loss-of-function variants or copy number variation deletions inducing recessive diseases [12]. In another study, mRNA targets of miRNAs were found to be significantly enriched in those genes with highly stable expression in response to external perturbations, thus validating the hypothesis that miRNAs can buffer mRNA expression fluctuation [13].

The above studies encouraged us to further investigate the importance of stable gene expression in a postnatal healthy untreated animal model. We hypothesized that there are genes whose expression is relatively stable across various organs, ages, and sexes and that these genes are involved in core cellular processes indispensable for survival and growth [14]. A large dataset of gene expression measurements of various organs across the lifespan and in both sexes from a well-controlled model system is required to thoroughly and quantitatively test this hypothesis. The rat has been extensively used by the academic community and pharmaceutical industry as an animal model to identify gene functions, evaluate drug responses and understand human diseases. Recently, Yu et al. [15] used RNA-seq to comprehensively profile gene expression levels in 11 organs at 4 ages (2wk, immature; 6 wk, adolescence; 21 wk, adult; and 104 wk, aged) and both sexes of the untreated Fischer 344 rat. The organs include representatives of most of the major organ systems, including the nervous system (brain), respiratory system (lungs), cardiovascular system (heart), digestive system (liver), endocrine system (adrenal glands), lymphatic system (thymus and spleen), excretory system (kidney), muscular system (muscle), and reproductive system (testes and uterus). There were four biological replicates per organ, age, sex combination resulting in 320 samples. An annotated database has been created as a web-based open-access resource (The Rat BodyMap database; http://pgx.fudan.edu.cn/ratbodymap/) and we have used it to identify stably expressed genes (SEGs). Additional large-scale genomics databases derived from mouse and human were used to explore and confirm the biological importance of these SEGs, including pathway analysis, cross-species consistency, effects of gene knock-out, and extent of disease-causing mutations. Observing the action of their homologs in other biological systems enabled us to elucidate the biological importance of these SEGs. The pathways involving SEGs were found to be core processes, which are important to maintain basic cellular functions. Tight control of these core processes is essential to prevent damage to cells, and any perturbation by endogenous (e.g., genetics) or exogenous agents (e.g., drugs, environmental factors) may cause serious adverse effects.

## Materials and Methods

### Rat tissue RNA-seq data

The normalized RNA-sequencing data of 320 organ samples (as measured by fragments per kilobase of transcript per million mapped reads, FPKM) from male and female rats of 4 ages (2, 6, 21 and 104 weeks) were downloaded from the Rat BodyMap website (http://pgx.fudan.edu.cn/ratbodymap/). The downloaded RNA-seq data consisted of 40064 transcript expression measurements for 11 organs (adrenal, brain, heart, kidney, liver, lung, muscle, spleen, testis, thymus and uterus). Transcripts without corresponding Entrez Gene ID and transcripts that were not expressed (i.e., FPKM value constantly equals zero) in all samples were excluded. For redundant transcripts (i.e., multiple transcripts corresponding to the same Entrez Gene ID), only the one with the highest average FPKM value across all samples was retained. The minimum non-zero FPKM value was substituted for all remaining zero values. A total of 19343 transcripts, each of which corresponded to a unique rat gene, were selected for subsequent analysis.

### Human tissue RNA-seq data

The raw RNA-sequencing data of human samples were retrieved from the NIH common fund's Genotype-Tissue Expression (GTEx) project [16] website (http://www.gtexportal.org/). The current version (V6) of GTEx contains transcriptomics data for 11983 organ samples from 570 subjects belonging to 6 different age brackets (20 yrs and older). The mode of death of these subjects is divided into 6 categories, of which only one category (violent and fast death) did not die of a disease process. Therefore, to avoid major pathological impact on gene expression, only the samples from subjects of violent and fast death were used in this study. To match the spectrum of human organs to the rat organs described above, only 10 types of human tissues were examined, i.e., adrenal gland, brain (cortex), heart (left ventricle), kidney (cortex), liver, lung, muscle (skeletal), spleen, testis and uterus (thymus is absent in adults). Sample characteristics are given in S1 Dataset. Following a procedure similar to the rat gene screening described above, those unnamed, non-expressed and redundant transcripts were excluded. A total of 47495 transcripts, each of which corresponded to a unique human gene, was selected for subsequent analysis.

### Genetic damage index

The Genetic Damage Index (GDI) values of 19558 genes were provided in the supplementary data of [17] and downloaded from the Proceedings of the National Academy of Sciences website (http://www.pnas.org/content/112/44/13615/suppl/DCSupplemental). This data was derived from all alleles with a minor allele frequency less than 0.5 in the 1000 Genomes Project [18, 19] and GDI represents the mutational damage in each protein coding gene accumulated in the general human population.

### Phenotype information of knockout mouse model

The phenotype information of knockout mouse models was collected from the International Mouse Phenotyping Consortium [20, 21] website (http://www.mousephenotype.org/). A total of 1357 mouse genes were categorized into genes observed or not observed to cause ‘complete preweaning lethality’ in knockout mice.

### Pathways from KEGG (Kyoto Encyclopedia of Genes and Genomes)

The association between rat genes and KEGG pathway annotations was built by querying all the genes in ArrayTrack [22], an FDA-developed genomic tool for managing, analyzing, and interpreting gene expression data (http://www.fda.gov/ScienceResearch/BioinformaticsTools/Arraytrack/). Pathway analysis performed by ArrayTrack provided a tabular list of all the queried genes in corresponding KEGG pathways.

### Determination of SEGs

For each candidate gene, the expression values across 320 rat samples were examined for expression stability. To minimize the impact of extreme expression values caused by random error, the highest and the lowest FPKM values were ignored. The expression fold change (FC) was then calculated as

$$FC=\frac{{fpkm}_{max-1}}{{fpkm}_{min-1}}$$

where *fpkm*_*max*−1_ and *fpkm*_*min*−1_ represented the second highest and the second lowest expression value across 320 samples for each gene, respectively. Those genes showing FC ≤4.0 were defined as SEGs in the Rat BodyMap model. All the rat SEGs were mapped to human and mouse homolog genes according to the homology groups retrieved from the HomoloGene database (http://www.ncbi.nlm.nih.gov/homologene) of the National Center for Biotechnology Information (NCBI).

### Statistical tests

A non-parametric Mann-Whitney U test (also known as Wilcoxon rank-sum test) was performed to examine the association between (1) the expression stability of rat genes and human homolog genes, (2) the expression stability of rat genes and the Genetic Damage Index of human homolog genes. All the human genes were ranked by the statistic of interest (i.e., expression stability in GTEx data or Genetic Damage Index). The null hypothesis was that the ranks of the homolog genes of rat SEGs were not significantly different from those of other genes. A p-value was calculated to determine whether the null hypothesis was valid.

In addition, Fisher’s exact test was performed to determine whether the SEGs were enriched in a certain category (i.e., a KEGG pathway or the genes causing complete preweaning lethality in mouse knockout model). The statistical question was framed in terms of the following 2 × 2 contingency table:

**Table:** 

	SEG Homologs	Other Genes
In category	*N*_*a*_	*N*_*c*_
Not in category	*N*_*b*_	*N*_*d*_

*N*_*a*_ and *N*_*b*_ represented the numbers of SEG homologs in or not in the category. *N*_*c*_ and *N*_*d*_ were the numbers of other (non-SEG) genes in or not in the category. The odds ratio (OR) was calculated as OR = (*N*_*a*_ × *N*_*d*_)/(*N*_*b*_ × *N*_*c*_). The null hypothesis was formulated as:

$$H_{0}:{Prop}_{1}={Prop}_{2}$$,

where *Prop*_*1*_ = *N*_*a*_ */ (N*_*a*_ *+ N*_*b*_*)* and *Prop*_*2*_ = *N*_*c*_ */ (N*_*c*_ *+ N*_*d*_*)*. For KEGG pathway analysis, the crude p-values were adjusted due to testing on multiple pathways, so as to control the false discovery rate (FDR) under 5%.

## Results

### Identification of Stably Expressed Genes (SEGs)

**Fig 1:**
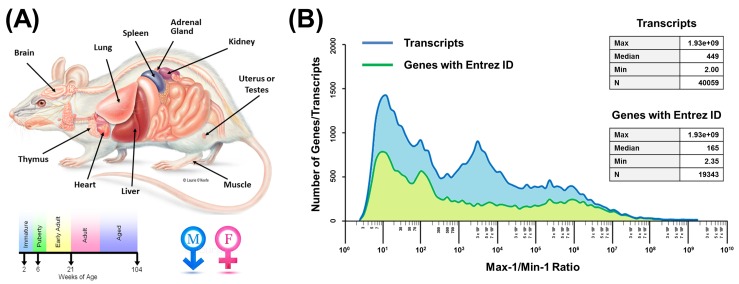
Study design and gene expression stability. (A) 320 organ samples were collected from rats of different ages and sexes. (B) A total of 19343 unique genes with Entrez ID were refined from 40059 transcripts. The distribution of expression changes demonstrated the rarity of genes with low variation across samples.

RNA-seq transcriptomics data were retrieved from the Rat BodyMap website. The samples consisted of 11 organs (adrenal gland, brain, heart, kidney, liver, lung, muscle, spleen, thymus, and testes or uterus) for 4 ages (2, 6, 21 and 104 weeks of age) and both sexes (Fig 1A). For each combination of age, sex and organ, there were 4 rats as biological replicates, resulting in 320 total samples. After excluding unnamed (without Entrez ID), non-expressed and redundant transcripts (see Materials and Methods), a total of 19343 unique genes (each corresponding to one transcript) remained for analysis of expression change in rats. The expression change for each gene was calculated as the maximum expression divided by the minimum expression for each gene across all sample. Initial inspection of the gene expression database showed an occasional expression value for some genes that was much higher or much lower than the other 320 samples (S1 Fig), suggesting that these outlier values may be technical artifacts of the measurement method. Removal of such values may allow better estimation of the expression change. Because this method of calculating gene expression change is sensitive to extreme outliers, the highest and lowest expression values of each gene were, therefore, removed before forming the ratio (see next paragraph for additional explanation). The distribution of the expression changes for these 19343 genes across these 320 samples is shown in Fig 1B (green), and ranged from 2.35 to >10^9^-fold, with a median of 165-fold. The expression changes of the complete set of 40059 transcripts (excluding 5 genes not expressed in any sample) is shown in Fig 1B (blue), and ranged from 2.0 to >10^9^-fold, with a median of 449. The distributions appear similar with the exception of a large peak of transcripts with expression changes of 1000–10000-fold in the complete transcript set.

**Fig 2:**
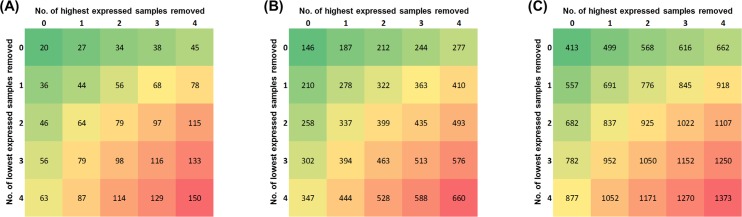
Numbers of stably expressed genes at different thresholds of expression fold-change and numbers of highest and lowest expressed samples removed. (A) Cutoff criteria of expression changes of ≤3-fold. (B) Cutoff criteria of expression changes of ≤4-fold. (C) Cutoff criteria of expression changes of ≤5-fold.

Our primary interest was identifying those genes showing relatively low expression variability across 11 organs, 4 ages, and both sexes. Two criteria were explored to set the cut-off threshold: (1) the ratio of the highest expression level of a gene to its lowest expression level (expression change) across all samples and (2) the number of highest and lowest expressing samples to be excluded. Adjusting these 2 factors allowed the selection of an appropriately low level of expression change with minimal interference by possibly false high or low expression values. Fig 2 shows matrix tables for the number of genes with expression changes of ≤3-fold, ≤4-fold, and ≤5-fold with removal of from 0–4 highest and lowest expression values. There were 20, 146 and 413 genes whose expression change was ≤3-fold, ≤4-fold, and ≤5-fold, respectively, and this number could be substantially increased by removing small numbers of highest and lowest expression values. As a trade-off for identifying genes with low expression variability without removing excessive numbers of samples, we focused on those genes whose expression varied by ≤4-fold after removal of the single highest and single lowest expressing samples. This resulted in 278 stably expressed genes (SEGs, S2 Dataset), which accounted for about 1% of all studied genes.

### Pathways enriched in SEGs

An unsupervised pathway analysis was performed to understand the associations between the 278 SEGs and Kyoto Encyclopedia of Genes and Genomes (KEGG) Pathway [23] terms (see Materials and Methods). One hundred six of these 278 SEGs were associated with at least one KEGG pathway (S2 Dataset). Pathways that were significantly enriched in SEGs included proteasome, ubiquitin mediated proteolysis, RNA transport, Epstein-Barr virus infection, aminoacyl-tRNA biosynthesis, protein processing in endoplasmic reticulum, legionellosis, and spliceosome (Table 1). The protein degradation pathway was notably represented with adjusted p-values of 1.8 x 10^−16^ and 6.4 x 10^−6^ for proteasome (16 genes) and ubiquitin mediated proteolysis (13 genes), respectively. And the odds ratio of SEGs enrichment was 41.52 for proteasome pathway and 8.01 for ubiquitin mediated proteolysis pathway. Most of the significant pathways were closely related to the processing of protein and RNA molecules, which are basic functions for all tissues and organs. The Epstein Barr virus infection pathway shared 10 genes (*Psmc4*, *Psmd11*, *Psmd13*, *Psmd3*, *Psmd6*, *Psmd4*, *Psmd12*, *Psmc1*, *Psmd1*, and *Psmd7*) with the proteasome pathway and 1 gene (*RGD1561926*) with the spliceosome pathway, suggesting that this pathway was significant because of the presence of protein degradation genes. The legionellosis pathway may have reached significance because of the presence of the genes *Sar1a* and *Vcp* that are also present in the protein processing in endoplasmic reticulum pathway.

**Table 1:** KEGG pathways significantly enriched in SEGs.

KEGG Pathway Term	SEGs Associated with the Pathway	OR	Adjusted P-value
Proteasome	Psmc4; Psma4; Psma3l; Psmd11; Psmd13; Psmb1; Psmd3; Psmd6; Psmd4; Psma1; Psmd12; Psmb4; Psmc1; Psmd1; Psmd7; Psma5	41.52	1.78 x 10^−16^
Ubiquitin mediated proteolysis	Ube2d3; Uba3; Cul1; Rbx1; Ube4a; Ube3c; Anapc5; Ddb1; Ube3a; Itch; Birc6; Keap1; Klhl9	8.01	6.41 x 10^−6^
RNA transport	Nmd3; Snupn; Eif3s10; Eif4g1; Eif3c; Elac2; Eif2b5; Eif4g2_predicted; Eif4b; Ranbp2; Eif3h	5.91	5.10 x 10^−4^
Epstein-Barr virus infection	Psmc4; RGD1561926; Psmd11; Psmd13; Psmd3; Psmd6; Psmd4; Psmd12; Psmc1; Polr2b; Psmd1; Psmd7	4.79	1.17 x 10^−3^
Aminoacyl-tRNA biosynthesis	Nars2; Farsb; Tars2; Zmat2; Sars; Lars	11.66	1.35 x 10^−3^
Protein processing in endoplasmic reticulum	Sar1a; Rad23b; Ube2d3; LOC685144; Nsfl1c; Cul1; Rbx1; Edem3; Dnajc10; Vcp	4.90	3.25 x 10^−3^
Legionellosis	Sar1a; Arf1; Sec22b; Rab1; Vcp	7.41	3.15 x 10^−2^
Spliceosome	Cwc15; Cdc5l; RGD1561926; Syf2; Prpf8; Plrg1; Prpf6	4.53	4.35 x 10^−2^

To understand the effect of changing the cutoff values on the types of genes and pathways identified, we adjusted the number of highest and lowest expressed genes to remove. Using an expression change of ≤4-fold and removing no samples, the highest and lowest expressed 2 samples, 3 samples, and 4 samples, resulted in 146, 399, 513, and 660 genes (along the diagonal in Fig 2B, FC≤4-fold panel). KEGG pathway analysis identified the same pathways as with the 278 SEGs, generally with increased significance level (Table 2; S1–S5 Tables). Examination of the significant pathways identified with cutoff criteria of expression changes of ≤3-fold (44 SEGs; S6 Table) and ≤5-fold (691 SEGs; S7 Table) calculated by using second highest and lowest samples (with exclusion of the single highest and single lowest expressed samples) also identified similar pathways involving protein degradation and processing, although with the expression change of ≤3-fold, only 17 of the 44 SEGs were associated with any KEGG pathway. Thus, our selection criteria appear to be robust and making small adjustments in them identify the same major pathways involved in protein degradation and processing.

**Table 2:** SEGs with expression changes of ≤4-fold–the number of genes in stably expressed pathways increases with relaxed selection criteria.

	Number of Genes in KEGG Pathways at Expression Change of ≤4-fold Calculated after Removal of N Highest and Lowest Expressed Samples					
KEGG Pathway Term	N = 0	N = 1	N = 2	N = 3	N = 4	Adjusted p-values
Proteasome	8	16	19	20	24	3.5 x 10^−7^–10^−21^
Ubiquitin mediated proteolysis	8	13	15	15	18	8.7 x 10^−3^–6.4 x 10^−6^
RNA transport	8	11	12	15	16	1.2 x 10^−3^–3 x 10^−4^
Aminoacyl-tRNA biosynthesis		6	8	9	15	1.4 x 10^−3^–3 x 10^−10^
Protein processing in endoplasmic reticulum		10	12	17	19	3 x 10^−3^–6 x 10^−5^
Spliceosome		7		12	13	4 x 10^−2^–2 x 10^−3^
Protein export			5	6	8	3 x 10^−3^–2 x 10^−5^
Legionellosis		5	6	6	7	>10^−2^
mTOR signaling pathway			6	8	8	>10^−2^
Nucleotide excision repair			5			>10^−2^
Renal cell carcinoma			6			>10^−2^
AMPK signaling pathway				10	11	>10^−2^
SNARE interactions in vesicular transport				5	6	>10^−2^
Insulin signaling pathway				10		>10^−2^
Regulation of autophagy					5	>10^−2^

### Expression stability of human homologs of SEGs

**Fig 3:**
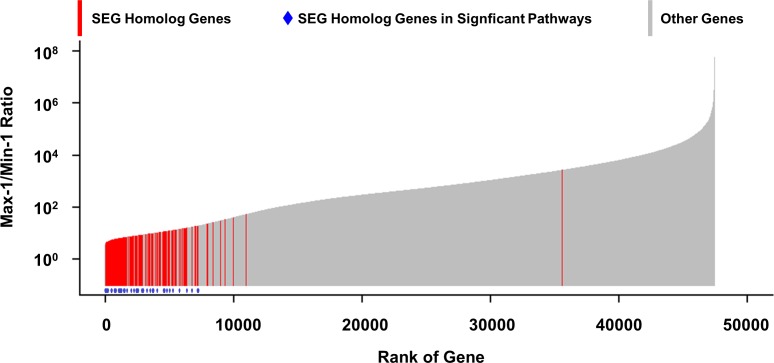
Expression stability of human homologs of SEGs. Each bar represented a gene, with the height corresponding to the expression change (maximum expression–minimum expression). The SEG homologs are red and those associated with significant pathways identified in Table 1 are marked with a blue dot.

The expression stability of these SEGs identified in the rat was examined in human tissues. The human homologs were identified and used to query the human RNA-sequence data retrieved from the Genotype-Tissue Expression (GTEx) database as described in Materials and Methods. Gene expression data was available for 98 human tissue samples from 10 tissue types that matched the rat (all except thymus) in both sexes and at ages from 20–69 years from individuals who did not die of a disease process (S1 Dataset). The expression variation was calculated for these genes (S3 Dataset; see Materials and Methods) across the 98 samples and is displayed in Fig 3. The expression fold change varied from 3.2 to 5.6 x 10^7^, with a median of 470. Those human genes homologous to the 278 rat SEGs are marked in red and the subset that fall into KEGG pathways are marked in blue in Fig 3. As can be verified by a non-parametric Mann-Whitney U test (see Materials and Methods), the human homologs of these rat SEGs are over-represented in the most stably expressed human genes with a highly significant p-value (2.49 x 10^−104^). Thus, the stability of expression of the rat SEGs is conserved across species, supporting the generalization of the findings.

### Lethality of SEG knockout

The cross-species consistency of SEGs suggested that expression stability of these genes may be of such importance that absence of the SEGs may result in major systemic disturbance and/or lethal outcomes. The International Mouse Phenotyping Consortium (IMPC) is producing knockout mouse strains for every protein coding gene and carrying out high-throughput phenotyping of each line [24], which enabled us to examine the association between SEG knockout and lethal phenotype (S4 Dataset; see Materials and Methods). Of the 1357 genes examined thus far by IMPC, 16 are homologous to the 278 rat SEGs and knocking out of 15 of these (94%) produced complete preweaning lethality, defined by IMPC as death anytime between fertilization and weaning age (approximately 3–4 weeks of age). Knockout of one of the genes (*Dnpep*) did not result in preweaning lethality but did result in a changed phenotype that included reduced T cell numbers. Of the remaining 1341 genes, knockout of 421 produced complete preweaning lethality (31%). Thus, absence of mouse genes homologous to the rat SEGs resulted in a significantly higher risk of lethal phenotype than absence of non-SEGs (P = 3.84×10^−7^; Table 3). Characteristics of the 15 SEGs with lethal knockout phenotypes are shown in Table 4. Thus, the strong association of SEGs with a lethal phenotype supports the interpretation of a fundamental role of SEGs in biological development.

**Table 3:** The association between SEGs knockout and lethal phenotypes.

	SEG Homologs	Other Genes
**Lethal Phenotypes Observed**	15	421
**Lethal Phenotypes Not Observed**	1	920
**Odds Ratio (95% CI)**	32.78 (4.32 ~ 248.97)	
**P-value**^**a**^	3.84 × 10^−7^	

^a^ P-value was determined with two-tailed Fisher’s exact test.

**Table 4:** Summary of SEGs with lethal knockout phenotypes.

Lethal Knockout Mouse Gene	Corresponding Rat SEG	Gene Name	NCBI Gene Summary
Alg10b	Alg10b	ALG10, alpha-1,2-glucosyltransferase	Regulatory component of non-inactivating K+ channels
Chd4	Chd4	Chromodomain helicase DNA binding protein 4	Member of the CHD protein family; may play a role in chromatin reorganization; may play a role in osmosignalling
Cog3	Cog3	Component of oligomeric golgi complex 3	
Eif4g2	Eif4g2 (predicted)	Eukaryotic translation initiation factor 4, gamma 2	This gene product functions as a general repressor of translation by forming translationally inactive complexes
Exoc8	Exoc8	Exocyst complex component 8	Encodes a subunit of the exocyst complex, an exocytosis-associated complex specifically located at sites of vesicle fusion
Lztr1	Lztr1	Leucine-zipper-like transcription regulator 1	
Maea	Maea	Macrophage erythroblast attacher	
Mapkap1	Mapkap1	Mitogen-activated protein kinase associated protein 1	
Ppp4r2	Ppp4r2	Protein phosphatase 4, regulatory subunit 2	
Raf1	Raf1	V-raf-leukemia viral oncogene 1	Acts as a mitogenic protein kinase; mutant forms may play a role in transformation
Rint1	RGD1560433	RAD50 interactor 1	
Sec22b	Sec22b	SEC22 homolog B, vesicle trafficking protein	
Usp36	Usp36	Ubiquitin specific peptidase 36	
Usp5	Usp5	Ubiquitin specific peptidase 5	
Vps13d	Vps13d	Vacuolar protein sorting 13 homolog D	

### Genetic damage index of SEGs

**Fig 4:**
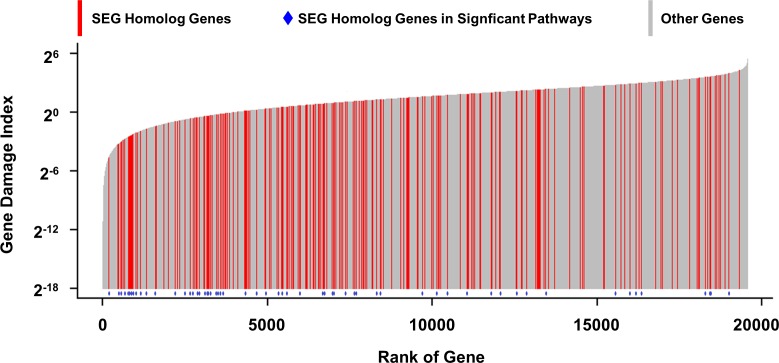
Human homolog genes of SEGs showed significantly lower Genetic Damage Index (GDI). Each bar represented a gene, with the height corresponding to the GDI value. The SEG homologs are red and those associated with significant pathways identified in Table 1 are marked with a blue dot.

Recently, the frequency of non-synonymous mutations in each protein coding gene of the general human population was examined and a metric was developed (gene damage index, GDI) to quantify the mutational damage accumulated for each gene [17]. The GDI was proposed as a genome-wide and gene-level metric of evolutionarily accumulated mutational damage, with higher values associated with a higher non-synonymous mutational load. Because non-synonymous DNA mutations can have profound impact on the function and expression of human protein-coding genes by changing the DNA codon and correspondent amino acid sequences, it would be expected that SEGs would have lower GDI scores than non-SEGs. GDI scores for human genes (S5 Dataset) are displayed from lowest to highest in Fig 4 and the homologs of the rat SEGs are highlighted in red. While the overall average GDI was 4.35 (ranging from 0 to 42.91), the average GDI of SEG homologs was lower at 3.25 (ranging from 0.04 to 19.08). Using a non-parametric Mann-Whitney U test, the ranks of human homologs of the rat SEGs were compared with those of other genes (see Materials and Methods) and found to be significantly (p = 1.92×10^−5^) different. It was noted by Itan et al [17] that genes with low GDI were found to be enriched in proteasome and spliceosome pathways, which were also highlighted in our KEGG pathways analysis on SEGs (as shown in Table 1). Thus, SEGs appear to be subjected to less DNA sequence variation than the bulk of protein coding genes suggesting that expression stability is reflected in evolutionary DNA sequence stability in this set of genes.

## Discussion

In the present study, we identified genes whose expression varied within a relatively narrow range across the life span, and between sexes and organs in F344 rats. The proteins encoded by these SEGs were found to play important roles in basic cellular functions, including protein catabolism (proteasome and ubiquitination), RNA transport, protein processing, and the spliceosome, and suggested stable expression of these genes is fundamental to good health. Several lines of evidence support the validity of these findings. First, the low expression variability of these genes was conserved across species. Examination of the expression of the human homologs of the rat SEGs in a database of tissues derived from humans (GTEx) showed that these genes exhibited much less expression variability across tissue, age and sex than other genes (p< 2.5 x 10^−104^). Second, we hypothesized that the transcriptomic stability of the SEGs played a pivotal role in normal physiological function and that dysregulation would result in disease or lethality. Examination of the effect of knocking out individual SEGs in mouse models developed in the International Mouse Phenotyping Consortium showed a lethal phenotype at the prenatal stage for a high proportion of SEGs. Of the 16 strains with knock-out of the mouse homolog of a rat SEG, 15 resulted in complete preweaning lethality (>93%); knocking out the mouse homologs of rat non-SEGs (1341 genes) resulted in complete preweaning lethality only 31% of the time, supporting the importance of these SEGs in development (p< 3.8 x 10^−7^). Third, we hypothesized that SEGs would be under evolutionary pressure to maintain stable expression and this would be reflected in the accumulation of fewer mutations in these genes. Recently, a human gene damage index (GDI) summarizing the non-synonymous mutational load of each protein-coding gene in the general human population was developed [17]. Examination of this database showed that the human homologs of the rat SEGs were less frequently mutated in healthy populations and exhibited lower GDI (p = 1.92×10^−5^). Thus, from multiple independent perspectives, stably expressed genes encoding proteins involved in protein catabolism, RNA transport, protein processing, and the spliceosome, appear to be of fundamental importance.

KEGG pathways analysis was performed to further interpret the pathological and pharmacological importance of SEGs. The results indicated that these genes were significantly involved in protein catabolism, such as proteasome components and ubiquitination. The ubiquitin-proteasome system degrades intracellular proteins in a highly regulated manner and is essential to a wide range of cellular processes [25]. Previous studies have shown that dysregulation and instability of the ubiquitin-proteasome system can result in multiple diseases [26], including aging [27]. For instance, elevated proteasomal activity has been detected in many types of cancers [28]. Because tumor growth and division can be halted by tumor suppressor proteins [29], tumor cells rely heavily on proteasomal function for the degradation of suppressor proteins [30]. Another example is the overactivity of the ubiquitin-proteasome system in rheumatoid arthritis. As a master regulator of many inflammatory cytokines involved in the pathogenesis of rheumatoid arthritis [31], NF-κB protein is normally inhibited when bound to IκB protein [32]. However, in the case of excessive ubiquitination of IκB followed by proteasomal degradation, NF-κB can be chronically active to cause inflammatory arthritis [33].

Since the 278 SEGs identified in our analysis are highly enriched in the ubiquitin-proteasome system, this may provide an opportunity to discover novel disease liability genes among SEGs. First, the expression levels can be monitored in disease models to confirm whether the expression stability of a certain SEG is maintained or altered in pathological conditions. Second, by using knockdown [34] or overexpression [35] techniques, gene expression can be purposely reduced or increased in particular age, sex or organ, to identify potential disease liabilities. It can then be determined if restoring the stability of expression will lead to beneficial outcomes in such disease models (e.g., suppression of tumor growth or anti-inflammatory effect). Third, because gene expression is an indirect measurement of biological function, direct examination of protein levels and pathway functions [36] are needed to confirm the biological significance of these findings. Finally, there have been wide discussions regarding the selection of stable reference genes for quantitative real-time PCR (qRT-PCR) [37]. The SEGs described here may offer better stable candidate reference genes for such pRT-PCR studies. Further validation of the proposed SEGs will be important for improving the accuracy of qRT-PCR.

Taken together, we identified a set of genes with a relatively narrow range of expression change across organs throughout the life span and between the sexes in a rat model. The proteins encoding by these genes are significantly involved in a series of housekeeping functions, including protein catabolism, RNA transport, protein processing, and the spliceosome. The fundamental biological importance of the stable expression of these genes was supported by multiple external data sources, including human organ transcriptomics, mouse knockout models, and human protein-coding gene mutations. Additional exploration of such stable gene expression, at the protein and pathway level, as well as in other gene expression datasets (including microarray data), may provide opportunities for disease gene identification, as well as potential drug targets for restoration of function.

## Supporting Information

S1 Dataset The information of GTEx samples used for analysis. (XLSX)

S2 Dataset 278 SEGs identified from Rat BodyMap. (XLSX)

S3 Dataset Expression fold changes of SEG homologs in human GTEx data. (XLSX)

S4 Dataset Genes causing complete preweaning lethality in mouse knockout models. (XLSX)

S5 Dataset GDI value adopted from reference. (XLSX)

S1 Fig Examples of outliers in gene expression data. The expression value for each of the 320 samples is shown for four genes.
(PPTX)

S1 Table Significant KEGG pathways identified with cutoff criteria of expression changes of ≤4-fold and without exclusion of samples. (DOCX)

S2 Table Significant KEGG pathways identified with cutoff criteria of expression changes of ≤4-fold and with exclusion of the single highest and single lowest expressed genes. (DOCX)

S3 Table Significant KEGG pathways identified with cutoff criteria of expression changes of ≤4-fold and with exclusion of the 2 highest and single lowest expressed samples. (DOCX)

S4 Table Significant KEGG pathways identified with cutoff criteria of expression changes of ≤4-fold and with exclusion of the 3 highest and single lowest expressed samples. (DOCX)

S5 Table Significant KEGG pathways identified with cutoff criteria of expression changes of ≤4-fold and with exclusion of the 4 highest and single lowest expressed samples. (DOCX)

S6 Table Significant KEGG pathways identified with cutoff criteria of expression changes of ≤3-fold and with exclusion of the single highest and single lowest expressed samples. (DOCX)

S7 Table Significant KEGG pathways identified with cutoff criteria of expression changes of ≤5-fold and with exclusion of the single highest and single lowest expressed samples. (DOCX)
