# Supplementary material for: Stably Expressed Genes Involved in Basic Cellular Functions
Source: PLoS One. 2017 Jan 26;12(1):e0170813. doi: 10.1371/journal.pone.0170813 (PMC5268456; doi:10.1371/journal.pone.0170813)
Supplement: S1 Fig — The expression value for each of the 320 samples is shown for four genes. (PPTX) [file pone.0170813.s006.pptx]

## Slide 1
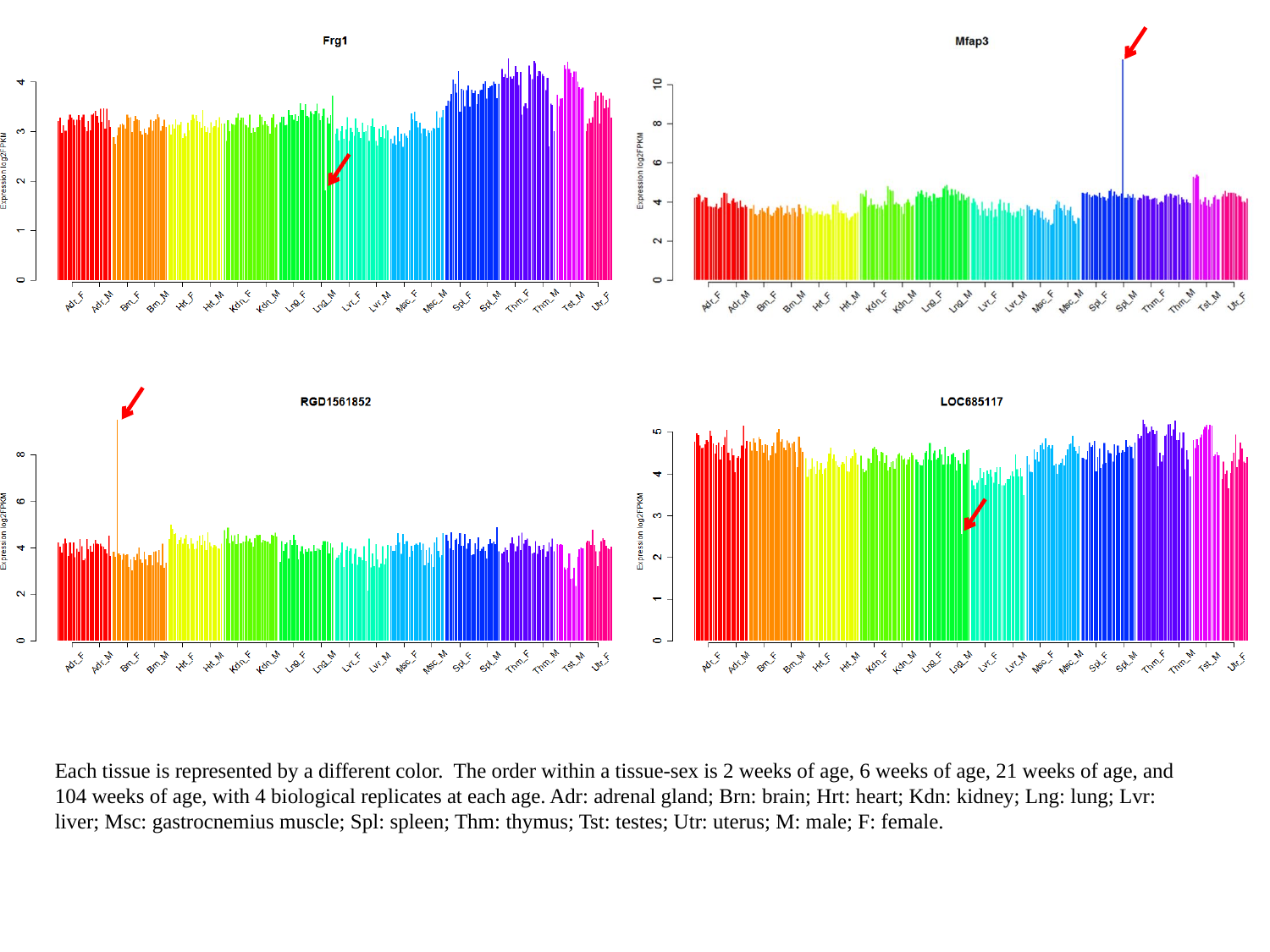

Each tissue is represented by a different color. The order within a tissue-sex is 2 weeks of age, 6 weeks of age, 21 weeks of age, and 104 weeks of age, with 4 biological replicates at each age. Adr: adrenal gland; Brn: brain; Hrt: heart; Kdn: kidney; Lng: lung; Lvr: liver; Msc: gastrocnemius muscle; Spl: spleen; Thm: thymus; Tst: testes; Utr: uterus; M: male; F: female.
