# Supplementary material for: Stably Expressed Genes Involved in Basic Cellular Functions
Source: PLoS One. 2017 Jan 26;12(1):e0170813. doi: 10.1371/journal.pone.0170813 (PMC5268456; doi:10.1371/journal.pone.0170813)
Supplement: S1 Table — (DOCX) [file pone.0170813.s007.docx]

| **KEGG Pathway Term** | **SEGs Associated with the Pathway** | **No. of genes (0)** | **OR** | **Adjusted P-value** |
| --- | --- | --- | --- | --- |
| Proteasome | Psmb1; Psmd6; Psmd4; Psma1; Psmb4; Psmd1; Psmd7; Psma5 | 8 | 30.86 | 3.49 x 10^7^ |
| Ubiquitin mediated proteolysis | Cul1; Ube3c; Anapc5; Ddb1; Itch; Birc6; Keap1; Klhl9 | 8 | 9.14 | 8.74 x 10^4^ |
| RNA transport | Eif4g1; Eif3c; Elac2; Eif2b5; Eif4g2_predicted; Eif4b; Ranbp2; Eif3h | 8 | 8.19 | 1.24 x 10^3^ |
