# Supplementary material for: Stably Expressed Genes Involved in Basic Cellular Functions
Source: PLoS One. 2017 Jan 26;12(1):e0170813. doi: 10.1371/journal.pone.0170813 (PMC5268456; doi:10.1371/journal.pone.0170813)
Supplement: S2 Table — (DOCX) [file pone.0170813.s008.docx]

| **KEGG Pathway Term** | **SEGs Associated with the Pathway** | **No of genes (1)** | **OR** | **Adjusted P-value** |
| --- | --- | --- | --- | --- |
| Proteasome | Psmc4; Psma4; Psma3l; Psmd11; Psmd13; Psmb1; Psmd3; Psmd6; Psmd4; Psma1; Psmd12; Psmb4; Psmc1; Psmd1; Psmd7; Psma5 | 16 | 41.52 | 1.78 x 10^16^ |
| Ubiquitin mediated proteolysis | Ube2d3; Uba3; Cul1; Rbx1; Ube4a; Ube3c; Anapc5; Ddb1; Ube3a; Itch; Birc6; Keap1; Klhl9 | 13 | 8.01 | 6.41 x 10^6^ |
| RNA transport | Nmd3; Snupn; Eif3s10; Eif4g1; Eif3c; Elac2; Eif2b5; Eif4g2_predicted; Eif4b; Ranbp2; Eif3h | 11 | 5.91 | 5.10 x 10^4^ |
| Epstein-Barr virus infection | Psmc4; RGD1561926; Psmd11; Psmd13; Psmd3; Psmd6; Psmd4; Psmd12; Psmc1; Polr2b; Psmd1; Psmd7 | 12 | 4.79 | 1.17 x 10^3^ |
| Aminoacyl-tRNA biosynthesis | Nars2; Farsb; Tars2; Zmat2; Sars; Lars | 6 | 11.66 | 1.35 x 10^3^ |
| Protein processing in endoplasmic reticulum | Sar1a; Rad23b; Ube2d3; LOC685144; Nsfl1c; Cul1; Rbx1; Edem3; Dnajc10; Vcp | 10 | 4.90 | 3.25 x 10^3^ |
| Legionellosis | Sar1a; Arf1; Sec22b; Rab1; Vcp | 5 | 7.41 | 3.15 x 10^2^ |
| Spliceosome | Cwc15; Cdc5l; RGD1561926; Syf2; Prpf8; Plrg1; Prpf6 | 7 | 4.53 | 4.35 x 10^2^ |
