# Supplementary material for: Stably Expressed Genes Involved in Basic Cellular Functions
Source: PLoS One. 2017 Jan 26;12(1):e0170813. doi: 10.1371/journal.pone.0170813 (PMC5268456; doi:10.1371/journal.pone.0170813)
Supplement: S3 Table — (DOCX) [file pone.0170813.s009.docx]

| **KEGG Pathway Term** | **SEGs Associated with the Pathway** | **No. of genes (2)** | **OR** | **Adjusted P-value** |
| --- | --- | --- | --- | --- |
| Proteasome | Psmc4; Psma4; Psma3l; Psmd11; Psmd13; Psmb1; Psmd3; Psmd6; Psmd4; Psma1; Psmd12; Psmb4; Psmc1; Psmd1; Psmd7; Psma5; Pomp; Psmb5; Psma2 | 19 | 37.84 | 1.54 x 10^18^ |
| Ubiquitin mediated proteolysis | Ube2d3; Uba3; Cul1; Rbx1; Ube4a; Ube3c; Anapc5; Ddb1; Ube3a; Itch; Birc6; Keap1; Klhl9; LOC680426; Cul2 | 15 | 6.45 | 6.71 x 10^6^ |
| Aminoacyl-tRNA biosynthesis | Nars2; Farsb; Tars2; Zmat2; Sars; Lars; Yars2; Rars | 8 | 11.38 | 1.39 x 10^4^ |
| Epstein-Barr virus infection | Psmc4; RGD1561926; Psmd11; Psmd13; Psmd3; Psmd6; Psmd4; Psmd12; Psmc1; Polr2b; Psmd1; Psmd7; Polr2f; Polr3f; Pik3ca | 15 | 4.19 | 5.67 x 10^4^ |
| RNA transport | Nmd3; Snupn; Eif3s10; Eif4g1; Eif3c; Elac2; Eif2b5; Eif4g2_predicted; Eif4b; Ranbp2; Eif3h; Rpp14 | 12 | 4.45 | 2.00 x 10^3^ |
| Protein export | Srp72; Srpr; Srp54a; Spcs2; Srp14 | 5 | 14.13 | 2.92 x 10^3^ |
| Protein processing in endoplasmic reticulum | Sar1a; Rad23b; Ube2d3; LOC685144; Nsfl1c; Cul1; Rbx1; Edem3; Dnajc10; Vcp; Atf6; Dnajb12 | 12 | 4.11 | 3.08 x 10^3^ |
| Legionellosis | Sar1a; Arf1; Sec22b; Rab1; Vcp; rCG_48149 | 6 | 6.27 | 2.15 x 10^2^ |
| mTOR signaling pathway | Cab39; Eif4b; Prkaa1; Tsc2; Pik3ca; RGD1311784 | 6 | 5.55 | 3.43 x 10^2^ |
| Nucleotide excision repair | Rad23b; Rbx1; Ddb1; Cdk7; Ccnh | 5 | 6.86 | 3.43 x 10^2^ |
| Renal cell carcinoma | Rbx1; Raf1; Crkl; Crk; Cul2; Pik3ca | 6 | 4.97 | 4.82 x 10^2^ |
