# Supplementary material for: Stably Expressed Genes Involved in Basic Cellular Functions
Source: PLoS One. 2017 Jan 26;12(1):e0170813. doi: 10.1371/journal.pone.0170813 (PMC5268456; doi:10.1371/journal.pone.0170813)
Supplement: S4 Table — (DOCX) [file pone.0170813.s010.docx]

| **KEGG Pathway Term** | **SEGs Associated with the Pathway** | **No of genes (3)** | **OR** | **Adjusted P-value** |
| --- | --- | --- | --- | --- |
| Proteasome | Psmc4; Psma4; Psma3l; Psmd11; Psmd13; Psmb1; Psmd3; Psmd6; Psmd4; Psma1; Psmd12; Psmb4; Psmc1; Psmd1; Psmd7; Psma5; Pomp; Psmd2; Psmb5; Psma2 | 20 | 31.79 | 5.72 x 10^18^ |
| Protein processing in endoplasmic reticulum | Sar1a; Rad23b; Ube2d3; LOC685144; Nsfl1c; Cul1; Rbx1; Edem3; Dnajc10; Vcp; Sec13; Dnaja2; Atf6; Ddost; Dnajb12; Ssr1; Sec62 | 17 | 4.68 | 7.05 x 10^5^ |
| Aminoacyl-tRNA biosynthesis | Nars2; Farsb; Tars2; Zmat2; Sars; Lars; Dars; Yars2; Rars | 9 | 10.17 | 8.82 x 10^5^ |
| Ubiquitin mediated proteolysis | Ube2d3; Uba3; Cul1; Rbx1; Ube4a; Ube3c; Anapc5; Ddb1; Ube3a; Itch; Birc6; Keap1; Klhl9; LOC680426; Cul2 | 15 | 4.95 | 9.79 x 10^5^ |
| RNA transport | Nmd3; Snupn; Eif3s10; Eif4g1; Eif3c; Elac2; Eif2b5; Eif4g2_predicted; Eif4b; Ranbp2; Eif3h; Rpp14; Eif3e; Sec13; Sap18 | 15 | 4.40 | 2.97 x 10^4^ |
| Protein export | Srp72; Srpr; Srp54a; Spcs2; Srp14; Sec62 | 6 | 13.92 | 7.15 x 10^4^ |
| Spliceosome | Cwc15; Cdc5l; RGD1561926; Syf2; Prpf8; Plrg1; Prpf6; RGD1565486; Siahbp1; Sf3b5; Hnrnpk; Xab2 | 12 | 4.35 | 1.75 x 10^3^ |
| Epstein-Barr virus infection | Psmc4; RGD1561926; Psmd11; Psmd13; Psmd3; Psmd6; Psmd4; Psmd12; Psmc1; Polr2b; Psmd1; Psmd7; Polr2f; Polr3f; Psmd2; Pik3ca | 16 | 3.45 | 1.75 x 10^3^ |
| mTOR signaling pathway | Cab39; Eif4b; Rps6kb1; Prkaa1; Tsc2; Frap1; Pik3ca; RGD1311784 | 8 | 5.95 | 3.59 x 10^3^ |
| AMPK signaling pathway | Cab39; Rab2a; Eef2; Elavl1; Rps6kb1; Prkaa1; Tsc2; Frap1; Pik3ca; RGD1311784 | 10 | 3.45 | 2.71 x 10^2^ |
| SNARE interactions in vesicular transport | Sec22b; Gosr1; Stx8; Vti1a; Use1 | 5 | 7.12 | 2.71 x 10^2^ |
| Insulin signaling pathway | Exoc7; Raf1; Crkl; Rps6kb1; Prkaa1; Crk; Tsc2; Frap1; Pik3ca; RGD1311784 | 10 | 3.15 | 4.17 x 10^2^ |
| Legionellosis | Sar1a; Arf1; Sec22b; Rab1; Vcp; rCG_48149 | 6 | 4.83 | 4.36 x 10^2^ |
