# Supplementary material for: Stably Expressed Genes Involved in Basic Cellular Functions
Source: PLoS One. 2017 Jan 26;12(1):e0170813. doi: 10.1371/journal.pone.0170813 (PMC5268456; doi:10.1371/journal.pone.0170813)
Supplement: S5 Table — (DOCX) [file pone.0170813.s011.docx]

| **KEGG Pathway Term** | **SEGs Associated with the Pathway** | **No. of genes (4)** | **OR** | **Adjusted P-value** |
| --- | --- | --- | --- | --- |
| Proteasome | Psmc4; Psma4; Psma3l; Psmd11; Psmd13; Psmb1; Psmd3; Psmd6; Psmd4; Psma1; Psmd12; Psmb4; Psmc1; Psmd1; Psmd7; Psma5; Pomp; Psmd2; Psmc6; Psmb7; Psmb3; Psmb5; Psma2; Psmd14 | 24 | 35.21 | 1.09 x 10^21^ |
| Aminoacyl-tRNA biosynthesis | Nars2; Farsb; Tars2; Zmat2; Sars; Lars; Wars; Gars; Nars; Lars2; Dars; Yars2; Dnd1; Rars; Mars | 15 | 16.07 | 3.37 x 10^10^ |
| Ubiquitin mediated proteolysis | Ube2d3; Uba3; Cul1; Rbx1; Ube4a; Ube3c; Anapc5; Ddb1; Ube3a; Itch; Birc6; Keap1; Klhl9; Anapc2; Anapc11; Uba1; LOC680426; Cul2 | 18 | 4.69 | 2.29 x 10^5^ |
| Protein export | Srp72; Srpr; Srp54a; Spcs2; Oxa1l; Srp14; Srp68; Sec62 | 8 | 16.36 | 2.29 x 10^5^ |
| Protein processing in endoplasmic reticulum | Sar1a; Rad23b; Ube2d3; LOC685144; Nsfl1c; Cul1; Rbx1; Edem3; Dnajc10; Vcp; Sec13; Dnaja2; Atf6; Amfr; Ddost; Dnajb12; Ssr1; Plaa; Sec62 | 19 | 4.07 | 6.28 x 10^5^ |
| RNA transport | Nmd3; Snupn; Eif3s10; Eif4g1; Eif3c; Elac2; Eif2b5; Eif4g2_predicted; Eif4b; Ranbp2; Eif3h; Eif2b1; Rpp14; Eif3e; Sec13; Sap18 | 16 | 3.63 | 9.78 x 10^4^ |
| Epstein-Barr virus infection | Psmc4; RGD1561926; Psmd11; Psmd13; Psmd3; Psmd6; Psmd4; Psmd12; Psmc1; Polr2b; Psmd1; Psmd7; Polr2f; Polr3f; Psmd2; Psmc6; Polr3a; Pik3ca; Psmd14 | 19 | 3.21 | 9.78 x 10^4^ |
| Spliceosome | Cwc15; Cdc5l; RGD1561926; Syf2; Prpf8; Plrg1; Prpf6; RGD1565486; Siahbp1; Sf3b5; Ddx23; Hnrnpk; Xab2 | 13 | 3.66 | 4.31 x 10^3^ |
| SNARE interactions in vesicular transport | Sec22b; Gosr1; Stx8; Vti1a; Ykt6; Use1 | 6 | 6.85 | 1.30 x 10^2^ |
| mTOR signaling pathway | Cab39; Eif4b; Rps6kb1; Prkaa1; Tsc2; Frap1; Pik3ca; RGD1311784 | 8 | 4.57 | 1.64 x 10^2^ |
| Legionellosis | Sar1a; Arf1; Sec22b; Rab1; Vcp; Hbs1l; rCG_48149 | 7 | 4.44 | 3.74 x 10^2^ |
| Regulation of autophagy | Becn1; Pik3c3; Prkaa1; Atg4b; LOC365601 | 5 | 6.48 | 3.74 x 10^2^ |
| AMPK signaling pathway | Cab39; Rab2a; Eef2; Elavl1; Rps6kb1; Prkaa1; Tsc2; Frap1; Pik3ca; Rab14; RGD1311784 | 11 | 2.94 | 4.21 x 10^2^ |
