# Supplementary material for: Stably Expressed Genes Involved in Basic Cellular Functions
Source: PLoS One. 2017 Jan 26;12(1):e0170813. doi: 10.1371/journal.pone.0170813 (PMC5268456; doi:10.1371/journal.pone.0170813)
Supplement: S6 Table — (DOCX) [file pone.0170813.s012.docx]

| **KEGG Pathway Term** | **SEGs Associated with the Pathway** | **OR** | **Adjusted P-value** |
| --- | --- | --- | --- |
| Ubiquitin mediated proteolysis | Cul1; Ube3c; Anapc5; Itch | 15.34 | 3.20 x 10^-2^ |
| Legionellosis | Sar1a; Rab1; Vcp | 28.75 | 3.20 x 10^-2^ |
| Protein processing in endoplasmic reticulum | Sar1a; Nsfl1c; Cul1; Vcp | 12.77 | 3.89 x 10^-2^ |
