# Supplementary material for: Stably Expressed Genes Involved in Basic Cellular Functions
Source: PLoS One. 2017 Jan 26;12(1):e0170813. doi: 10.1371/journal.pone.0170813 (PMC5268456; doi:10.1371/journal.pone.0170813)
Supplement: S7 Table — (DOCX) [file pone.0170813.s013.docx]

| **KEGG Pathway Term** | **SEGs Associated with the Pathway** | **OR** | **Adjusted P-value** |
| --- | --- | --- | --- |
| Proteasome | Psmc4; Psma4; Psma3l; Psmd11; Psmd13; Psmb1; Psmd3; Psmd6; Psmd4; Psma1; Psmd12; Psmb4; Psmc1; Psmd1; Psmd7; Psma5; Pomp; Psmd2; Psmc6; Psmb2; Psmb7; Psmb5; Psma2 | 30.55 | 1.03 x 10^-19^ |
| Ubiquitin mediated proteolysis | Ube2d3; Uba3; Cul1; Rbx1; Ube4a; Ube3c; Anapc5; Ddb1; Ube3a; Itch; Birc6; Keap1; Klhl9; Anapc2; Anapc11; Anapc4; RGD1563853; Uba1; LOC680426; Cul2 | 5.07 | 1.82 x 10^-6^ |
| Protein export | Srp72; Srpr; Srp54a; Sec63; Spcs2; Oxa1l; Srp14; Srp9; Sec62 | 18.92 | 1.82 x 10^-6^ |
| Spliceosome | Cwc15; Cdc5l; RGD1561926; Syf2; Prpf8; Plrg1; Prpf6; RGD1565486; Siahbp1; Sf3b5; Prpf4; Sf3b1; Ddx23; Usp39; Hnrnpk; Sart1; Xab2; LOC679898; RGD1307882 | 5.47 | 1.82 x 10^-6^ |
| Protein processing in endoplasmic reticulum | Sar1a; Rad23b; Ube2d3; LOC685144; Nsfl1c; Cul1; Rbx1; Edem3; Dnajc10; Vcp; Sec63; Sec13; Rpn2; Dnaja2; Atf6; Amfr; Ddost; Dnajb12; Mbtps1; Ssr1; Sec62 | 4.36 | 6.75 x 10^-6^ |
| Aminoacyl-tRNA biosynthesis | Nars2; Farsb; Tars2; Zmat2; Sars; Lars; Gars; Dars; Yars2; Rars; Mars | 9.72 | 7.08 x 10^-6^ |
| RNA transport | Nmd3; Snupn; Eif3s10; Eif4g1; Eif3c; Elac2; Eif2b5; Eif4g2_predicted; Eif4b; Ranbp2; Eif3h; Rpp14; Eif3e; Sec13; Clns1a; Tpr; Sap18; Strap | 3.96 | 1.26 x 10^-4^ |
| Epstein-Barr virus infection | Psmc4; RGD1561926; Psmd11; Psmd13; Psmd3; Psmd6; Psmd4; Psmd12; Psmc1; Polr2b; Psmd1; Psmd7; Polr2f; Polr3f; Psmd2; Psmc6; Polr3a; Pik3ca | 2.87 | 4.28 x 10^-3^ |
| Endocytosis | Arf1; Tsg101; Epn1; Vps37a; Chmp1b; Itch; Vps28; Cdc42; Arfgap2; Rufy1; Arfgef1; Rab5c; Tgfbr1; Arfgap1; Cltc; Vps24; Ap2a2; Rab11a | 2.66 | 9.43 x 10^-3^ |
| RNA degradation | Exosc4; Dhx36; RGD1564560; Pnpt1; Exosc9; Skiv2l2; Exosc7; RGD1306062; Cnot7 | 4.02 | 1.83 x 10^-2^ |
| mTOR signaling pathway | Cab39; Eif4b; Rps6kb1; Prkaa1; Tsc2; Frap1; Pik3ca; RGD1311784 | 4.36 | 2.05 x 10^-2^ |
| Renal cell carcinoma | Rbx1; Raf1; Crkl; Cdc42; Sos1; Crk; Cul2; Pik3ca | 3.89 | 3.67 x 10^-2^ |
| Regulation of autophagy | Becn1; Pik3c3; Prkaa1; Atg4b; LOC365601 | 6.17 | 4.29 x 10^-2^ |
| Nucleotide excision repair | Rad23b; Rbx1; Ddb1; Cdk7; RGD1563853; Ccnh | 4.80 | 4.64 x 10^-2^ |
